# From Reactive to Proactive Healthcare: Synergizing Wearable Biomarkers and Machine Learning in Digital Therapeutics

**DOI:** 10.3390/bioengineering13090977

**Published:** 2026-08-25

**Authors:** Kwanjoon Park, Eunice Kwan Chae Park, Woo Hyun Park, Eun-Young Jeon

**Affiliations:** 1 Department of Software Engineering, College of Engineering, Chonnam National University, Gwangju 61186, Republic of Korea; 213157@jnu.ac.kr; 2 Creative Technology Management, Humanities, Arts, and Social Sciences Division, Underwood International College, Yonsei University, Seoul 03722, Republic of Korea; eunicepark@yonsei.ac.kr; 3 Department of Physiology, Medical School, Jeonbuk National University, Jeonju 54896, Republic of Korea; 4 Institute of Humanities, Yonsei University, Seoul 03722, Republic of Korea

**Keywords:** biomedical informatics, digital therapeutics, machine learning pipelines, multimodal sensor fusion, digital biomarkers, health data privacy

## Abstract

The integration of digital therapeutics (DTx), wearable electronic devices, and artificial intelligence (AI) represents a significant advancement in personalized healthcare. The primary purpose of this structured narrative review is to evaluate the convergence of these technologies, providing a consolidated framework that bridges the gap between raw biometric data acquisition and actionable, AI-driven clinical insights. This paper synthesizes the latest literature on the intersection of mobile health (mHealth), machine learning (ML), and physiological tracking, with a primary focus on heart rate variability (HRV) and associated biochemical markers, such as cortisol, salivary alpha-amylase, and interleukins. Instead of viewing wearable outputs simply as raw data, we critically evaluate the technical verification and clinical validation required to define them as true “digital biomarkers.” By evaluating multimodal sensor technologies and advanced predictive algorithms, this paper outlines the clinical utility of digital biomarkers in diagnosing and proactively managing cardiovascular, neurological, metabolic, and psychiatric conditions, noting classification accuracies frequently exceeding 85% in controlled settings. However, we strongly caution that internally validated performance in controlled settings does not inherently demonstrate external clinical utility. The clinical relevance of this study lies in its holistic approach to identifying how continuous monitoring can broaden healthcare accessibility while improving precision medicine. Furthermore, it deeply addresses the technical challenges of highly variable ambulatory data quality, the necessity for robust artifact reduction (e.g., via LSTM and GAN architectures), and the limitations of small, homogeneous training datasets. We highlight the essential need for demographic-aware algorithmic models, external validation, and decentralized privacy-preserving models (e.g., federated learning) in diverse populations to ensure the safe, equitable clinical translation of DTx, mHealth, ML, and AI technologies.

## 1. Introduction

The transition from reactive clinical interventions to proactive, continuous health monitoring has been vastly accelerated by the advent of wearable biosensors and digital therapeutics (DTx). In a landmark analysis, Donnelly et al. [1] elucidated that continuous biomarker monitoring—initially popularized by continuous glucose monitoring (CGM) for diabetes management—is expanding rapidly to encompass continuous protein monitoring (CPM) for precision medicine. By capturing dynamic physiological and biochemical states via implantable or wearable technology, these platforms generate complex, longitudinal data streams capable of proactively tracking chronic metabolic, cardiovascular, and neurodegenerative diseases, offering the potential to enhance standard disease management protocols [1]. Furthermore, the urgency of advancing these sensing technologies is highlighted by Güntner et al. [2], who emphasize that tracking non-conventional biological matrices, such as interstitial fluid, sweat, tears, and breath, offers unprecedented longitudinal biomarker profiles outside traditional medical facilities.

**Figure 1:**
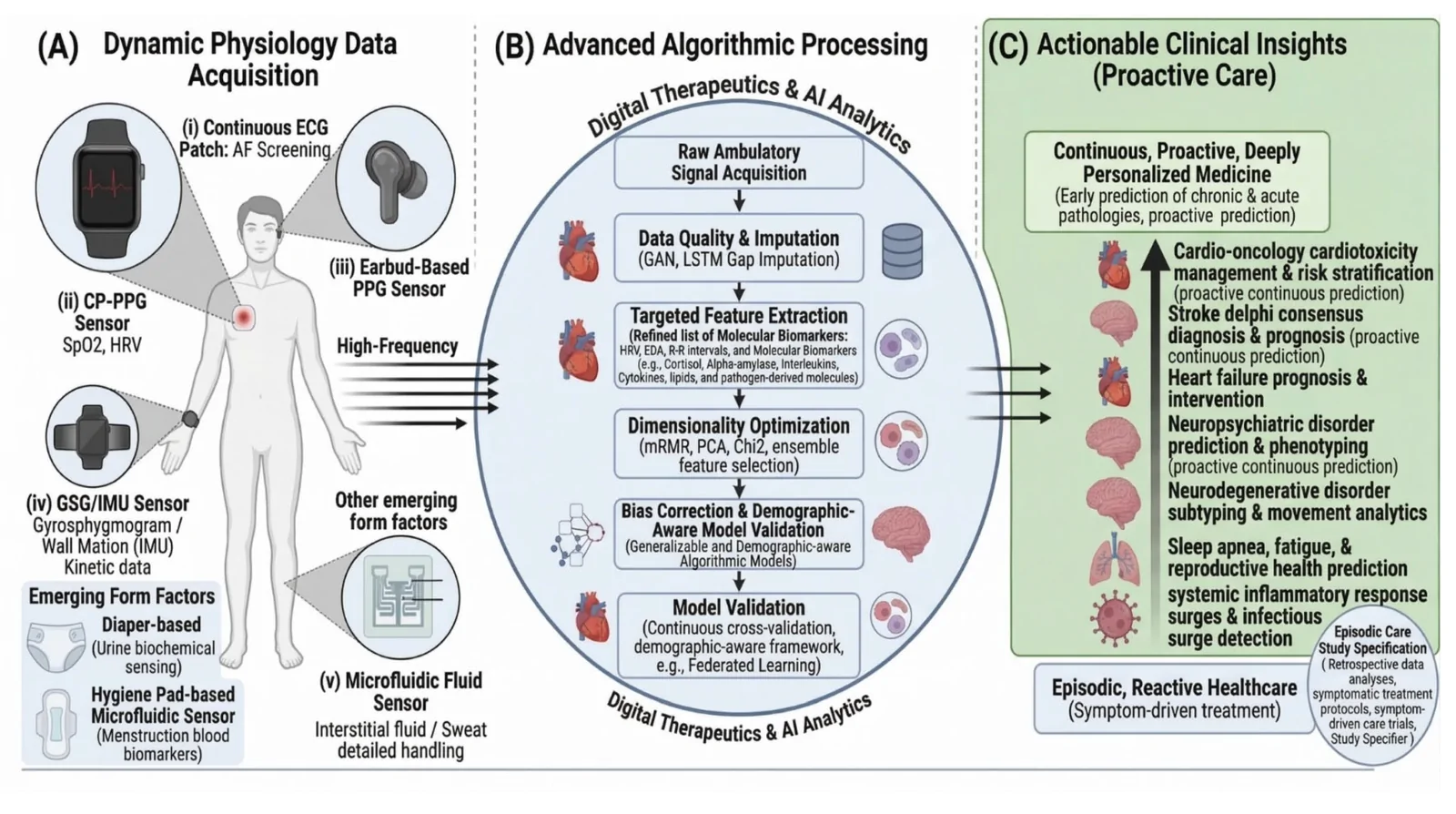
**Conceptual Framework: Proactive Healthcare.** An integrated platform converging wearable bio-acquisition, algorithmic processing, and proactive clinical outcomes. (**A**) **Dynamic Physiology Data Acquisition.** Biosensors (ECG patches, CP-PPG, GSG/IMU, microfluidics) continuously capture multi-modal physiological and biochemical data, transmitting raw ambulatory streams. (**B**) **Advanced Algorithmic Processing.** A central AI/ML pipeline executes data imputation (LSTMs), artifact correction (GANs), targeted feature engineering, and dimensionality optimization. Models are validated using demographic-aware frameworks (e.g., Federated Learning). (**C**) **Actionable Clinical Insights (Proactive Care).** Highlights the paradigm shift from traditional “Episodic, Reactive Healthcare” to “Continuous, Proactive, Highly Tailored Medicine,” targeting early detection across cardiovascular, neurological, metabolic, and infectious pathologies. The arrows indicate the continuous flow of data from raw acquisition to algorithmic processing, ultimately leading to proactive clinical interventions.

To visually conceptualize the intersection of these domains, please refer to Figure 1, which provides a comprehensive schematic of the dynamic data flow from ubiquitous physiological sensors to actionable clinical insights. However, the inherent complexity of pathophysiological processes necessitates precise, longitudinal biomarker tracking that current clinical data architectures often struggle to support. For instance, an international Delphi consensus recently reported that current clinical algorithms severely lack the precision to predict stroke progression or assess recurrence risk pre-hospitalization. To address this computational gap, 17 targeted research priorities and minimum reporting datasets were proposed to standardize stroke informatics and validate digital biomarkers [3]. Similarly, in adjacent medical fields ranging from anterior ocular inflammatory diseases to severe neurodevelopmental disorders like autism, DTx and artificial intelligence (AI)-assisted diagnostics are emerging as critical computational tools to supplement behavioral observations and bridge significant diagnostic gaps [4,5].

Digital biomarkers derived from smartwatches, fitness bands, and specialized biosensing patches capture immense, high-frequency data streams. Yet, raw data points alone lack clinical utility without the application of sophisticated machine learning (ML) and AI algorithms capable of filtering ambulatory noise and deciphering complex, non-linear physiological patterns [6]. Among the most vital indicators of autonomic nervous system function is heart rate variability (HRV), which has emerged as a cornerstone metric in mobile health (mHealth) applications ranging from dynamic stress detection to the early algorithmic prediction of atrial fibrillation (AF) and anxiety disorders [7,8].

It is crucial to clearly distinguish the terminology within this rapidly evolving field. While “digital health” encompasses broad wellness tracking, and “wearable sensing” refers merely to hardware data acquisition, “Digital Therapeutics (DTx)” are defined as evidence-based, software-driven interventions designed to prevent, manage, or treat a medical disorder, often operating in a closed-loop system. Furthermore, sensor-derived variables cannot automatically be deemed “digital biomarkers”; they must undergo rigorous technical verification (sensor reliability), analytical validation (algorithmic accuracy), and clinical validation (correlation with true health outcomes) prior to clinical implementation [9].

Therefore, the principal objective of this review is to critically examine the current state-of-the-art in wearable sensor technologies and multimodal digital biomarkers strictly from a biomedical informatics perspective. The scientific significance of this work lies in its ability to synthesize a highly fragmented landscape of data processing techniques into a cohesive informatics and algorithmic framework. By highlighting the latest advances in multi-sensor fusion architectures, ambulatory data artifact correction protocols, and explainable AI (XAI), this review aims to provide informaticians, data scientists, and bioengineers with a clear computational roadmap. Ultimately, it delineates how structured data pipelines and demographic-aware models can be utilized to transform noisy, high-dimensional physiological streams into reliable, generalizable digital phenotypes.

### Statement of Significance

The following table summarizes the core problems addressed, current knowledge gaps, and the unique contributions of this review.

**Category**

**Description**

**(1) Problem or Issue**
Translating raw wearable biosensor data into actionable clinical insights is heavily hindered by signal artifacts, algorithmic bias, and fragmented computational architectures in ambulatory settings.
**(2) What is Already Known**
While digital therapeutics (DTx) utilize machine learning to analyze physiological data (e.g., HRV), existing models often lack demographic-aware generalization, robust privacy protocols, and standardized informatics pipelines.
**(3) What this Paper Adds**
This review establishes a comprehensive biomedical informatics framework encompassing multimodal sensor fusion, deep learning-based artifact correction (e.g., GANs, LSTMs), and decentralized, privacy-preserving machine learning algorithms (e.g., federated learning).
**(4) Who would benefit**
Informaticians, data scientists, and clinical researchers developing equitable, generalizable, and AI-driven closed-loop predictive models for next-generation digital therapeutics.


## 2. Literature Search Strategy and Selection Criteria

To ensure the rigor of this structured narrative review, a literature search was conducted across major academic databases, including PubMed, IEEE Xplore, Scopus, and Web of Science. The search strategy employed Boolean operators combining keywords such as (“Wearable Sensors” OR “mHealth”) AND (“Machine Learning” OR “Deep Learning”) AND (“Digital Therapeutics” OR “Digital Biomarkers” OR “Multimodal Fusion”). The inclusion criteria were restricted to peer-reviewed journal articles primarily published between 2020 and 2026, focusing on clinical or ambulatory applications of sensor fusion and AI-driven physiological analysis. Conference proceedings, non-English publications, and studies lacking explicit validation metrics (e.g., accuracy, AUC, cross-validation details) were generally excluded to ensure data quality. Rather than performing a quantitative meta-analysis—which is often misleading due to extreme heterogeneity in AI model architectures and datasets—this review utilizes a critical synthesis approach. We specifically extracted data regarding sample sizes, model validation methodologies, and computational constraints to objectively evaluate the clinical translational potential and generalizability of each proposed algorithmic pipeline.

## 3. Wearable Sensor Technologies and Digital Biomarker Acquisition

Wearable electronic devices have evolved dramatically from simple accelerometers tracking steps to sophisticated, multimodal diagnostic platforms capable of capturing both biomechanical and biochemical data with clinical-grade accuracy [10]. Please refer to Table 1 for a summarized breakdown of the primary sensor modalities and their corresponding clinical targets.

### 3.1. Physiological Tracking (ECG and PPG)

The widespread adoption of consumer wearables has broadened access to continuous photoplethysmography (PPG) and electrocardiogram (ECG) data. Wrist-worn devices, smart rings, and in-ear sensors allow for the continuous, non-invasive extraction of heart rate (HR) and HRV parameters [13,24]. For instance, Santos et al. [13] demonstrated a real-time, single-ear wearable ECG reconstruction and R-peak detection system utilizing state-of-the-art in-ear electrode technology. Integrated with an optimized algorithm running on an energy-efficient device, this system yielded an exceptionally low mean error of 0.49 bpm for HR and 25.82 ms for HRV. Strikingly, the total system power consumption was merely 7.6 mW, enabling an estimated 36-h battery life suitable for continuous ambulatory monitoring [13]. Similarly, earbud-based PPG has been utilized via deep transfer learning to successfully predict HRV features for robust downstream stress classification, yielding at least a 5% improvement in accuracy and sensitivity compared to baseline state-of-the-art libraries [11].

Despite their ubiquity, PPG signals are highly susceptible to motion artifacts and suboptimal skin-sensor contact in ambulatory settings, which can severely distort signal morphology and degrade the accuracy of downstream physiological monitoring [12]. To counteract this, researchers have developed deep learning frameworks capable of transforming contact-pressure-distorted PPG signals into ideal morphologies, commonly referred to as contact-pressure mitigated PPG (CP-PPG). This adversarial deep generative model approach achieved a 40% improvement in signal fidelity (mean absolute error [MAE]: 0.09) and improved downstream HR and HRV estimation by 21% and 41–46%, respectively, highlighting the critical importance of mitigating skin-sensor contact issues [12]. Additionally, generative adversarial networks (GAN) with attention mechanisms (Att-SNGAN) have been employed to reconstruct highly accurate ECG signals directly from non-contact ballistocardiogram (BCG) signals collected via fiber optic sensors. This system achieved a minimal root mean square error (RMSE) of 0.0735 and a Fréchet Distance of 0.0342, offering new solutions for long-term continuous cardiac cycle monitoring at home [25]. Advanced inertial measurement units (IMUs) have also introduced novel modalities like the gyrosphygmogram (GSG) to complement traditional ECG and chest-based gyrocardiography. The GSG captures gyroscopic signals derived from arterial wall motion at the wrist, offering enhanced practicality and robustness to motion artifacts for continuous, real-time cardiovascular monitoring without reliance on traditional electrodes [15].

### 3.2. Biochemical and Microfluidic Sensors

Moving beyond traditional electrophysiological signals, next-generation wearables incorporate microfluidic systems for the real-time analysis of biofluids such as sweat, saliva, tears, and interstitial fluid [26]. These biochemical sensors enable the continuous monitoring of metabolic and stress-related biomarkers, including cortisol, glucose, lipid levels, and immune mediators [27,28,29]. Endrass et al. [29] highlighted that near-infrared, saliva-based, and smartphone-enabled fingertip devices show promising accuracy in tracking lipids to predict cardiovascular risk non-invasively. Wearable electrochemical biosensors have shown exceptional promise in monitoring the dynamic biochemical changes in chronic wounds by tracking pH, temperature, inflammatory cytokines, metabolites, and pathogen-derived molecules directly within the wound microenvironment, enabling closed-loop theranostics and AI-assisted decision-making [10]. Similarly, salivary biomarkers are being monitored for periodontal inflammation via a wearable molecularly imprinted polymer (MIP) sensor integrated into a mouthguard platform. Using a graphene oxide interlayer, this impedance-based mechanism achieves real-time, label-free detection of matrix metalloproteinase-8 (MMP-8), tracking charge transfer resistance changes via redox probes to identify disease progression accurately [18].

Advancements in passive sweat sensing—which utilizes nanoporous and cactus-inspired biomimetic Janus membranes to collect ultra-low sweat volumes—have eliminated the need for active sweat gland stimulation. By unidirectionally transporting and concentrating sweat toward a detection zone via Laplace pressure, these patches drastically improve biosafety and broad applicability by physically isolating chemical indicators from the epidermis to prevent allergic reactions or epidermal contamination [19,30]. Furthermore, utilizing discrete sweat sensing algorithms to compute both the number of active sweat glands and individual sweat rates has minimized error rates to below 0.2% for low and medium sweat volumes, significantly improving the precision of biomarker concentration estimates tailored to dynamic physiological output [31].

Highly innovative form factors are also emerging rapidly; Dosnon et al. [32] developed an electronic-free, microfluidic monitoring platform integrated into hygiene pads for the naked-eye and smartphone-assisted detection of disease biomarkers, such as C-reactive protein (CRP), carcinoembryonic antigen (CEA), and cancer antigen 125 (CA-125), in menstruation blood. Utilizing a ML smartphone algorithm, this platform reads biomarker-induced color changes, expanding women’s healthcare access with an affordable, non-invasive method [32]. Concurrently, Dong et al. [33] successfully designed a kilometer-scale wireless and wearable biochemical sensing platform integrated seamlessly into a diaper. Utilizing narrowband Internet of Things (NB-IoT), this platform continuously monitors urine biomarkers like dimethylamine, creatinine, glucose, and hydrogen ions across tens of kilometers, employing a multilayer perceptron data calibration system to reduce errors caused by varying pH levels, driving deep AI-wearable fusion [33].

## 4. Machine Learning Algorithms in Physiological Data Analysis

**Figure 2:**
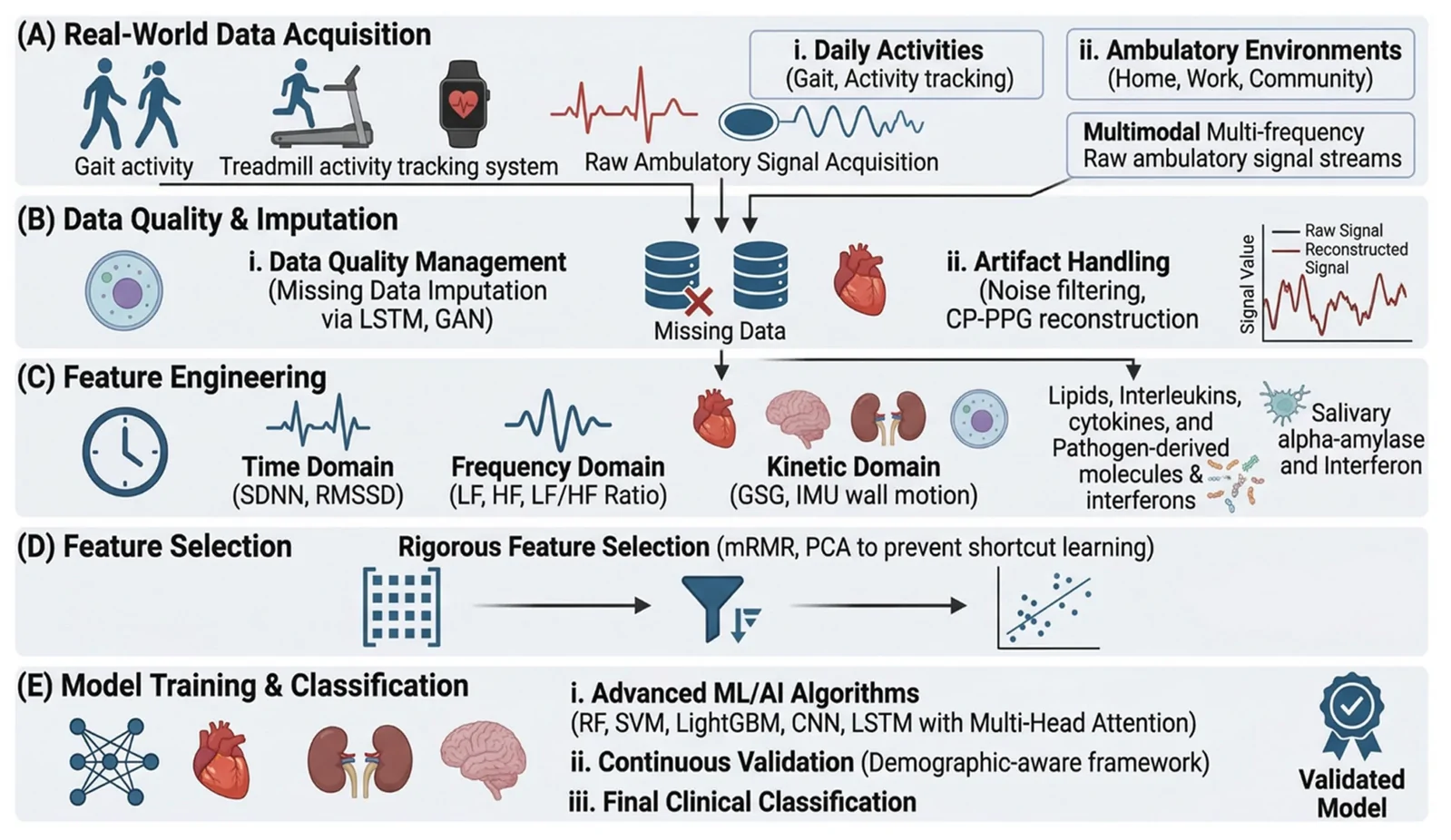
**Rigorous Machine Learning Pipeline for Physiological Data Analysis.** A five-stage systematic pipeline for processing ambulatory data: (**A**) **Real-World Data Acquisition.** Captures raw ambulatory signal streams from diverse wearables. (**B**) **Data Quality & Imputation.** Employs LSTM and GAN techniques for missing data imputation and adversarial networks for CP-PPG morphology reconstruction. (**C**) **Feature Engineering.** Extracts features across Time, Frequency, Kinetic, and Non-linear domains, representing multimodal biomarkers. (**D**) **Feature Selection.** Utilizes mRMR and PCA algorithms to optimize dimensionality and prevent shortcut learning. (**E**) **Model Training & Classification.** Final models are trained using advanced algorithms (RF, LightGBM, CNN, multi-head attention LSTMs) and validated via Federated Learning to generate equitable predictive insights across clinical domains. The arrows indicate the sequential flow of data processing, from raw signal acquisition to final model classification.

The massive volume of continuous, high-frequency data generated by wearable sensors necessitates highly robust analytical frameworks. Consequently, robust ML pipelines have become an essential cornerstone for addressing real-world challenges such as missing data imputation and artifact removal, alongside comprehensive multi-domain feature extraction and predictive modeling in the DTx space [34]. Figure 2 illustrates the systematic, step-by-step progression of this rigorous analytical framework, moving from ambulatory data acquisition to demographically balanced clinical model validation.

### 4.1. Algorithmic Approaches and Feature Selection

Commonly deployed algorithms include random forest (RF), support vector machines (SVM), light gradient boosting machines (LightGBM), and complex convolutional neural networks (CNN) [35,36]. A systematic review by Pataca et al. [35] analyzed literature from 2010 to 2025, revealing that ML models predicting stress episodes using multi-sensor data (electrodermal activity [EDA], PPG, accelerometers) consistently demonstrated high predictive accuracy, frequently reaching up to 99% in controlled environments. To improve computational efficiency on resource-constrained devices, researchers also leverage multitask learning (MTL) approaches that exploit shared underlying characteristics across tasks (e.g., simultaneously estimating HR and respiration rate while assessing signal quality), significantly reducing error rates and computational load compared to baseline single-task models [37].

The success of these models relies heavily on rigorous feature selection to eliminate redundant data and prevent overfitting. Methods such as minimum redundancy maximum relevance (mRMR) and principal component analysis (PCA) are frequently utilized to optimize dimensionality [24,38]. For example, in an extensive study aiming to predict driver drowsiness—a major cause of traffic fatalities resulting from fatigue and sleep deprivation—an RF classifier achieved a testing accuracy of 86.05%, precision of 87.16%, and a recall of 93.61% using precisely selected HRV features derived from PPG signals, proving its robustness for real-world integration into advanced driver assistance systems (ADAS) [39].

### 4.2. Handling Data Quality and Artifacts

Artifact correction remains a critical hurdle for the clinical adoption of digital biomarkers. Traditional filtering methods are often insufficient for real-world, ambulatory data. To prevent performance inflation and “shortcut learning,” advanced causal frameworks are strictly necessary to rigorously validate digital features as genuine biomarkers, particularly when interpreting complex, multi-source outputs like knee joint acoustical emissions [40]. Deep learning approaches, such as long short-term memory (LSTM) networks, are increasingly employed to reconstruct missing or distorted signals.

Interestingly, recent research indicates that utilizing adaptive LSTM models can accurately predict psychosis relapses in schizophrenia spectrum patients even when traditional artifact-removal preprocessing is entirely bypassed. Tsakmaki et al. [41] demonstrated that an LSTM model, optimizing sequence length for individual physiological patterns across 37 patients, achieved an extraordinary mean F1 score of 0.9817 on unprocessed HRV time series. This proved that omitting complex data cleaning steps (like wavelet and GAN-based denoising) did not reduce—and occasionally improved—prediction accuracy by leveraging the ML model’s ability to learn from raw, individualized temporal physiological trends directly [41]. Furthermore, the post-processing of R-wave detection utilizing Z-score normalization or min-max normalization combined with non-linear ML algorithms has vastly improved the reliability of HRV analysis in daily life environments, minimizing misdetected R-waves with a Matthews correlation coefficient (MCC) of 0.86 [42]. Data loss caused by poor sensor contact is also heavily mitigated through comprehensive data degradation testing to ensure reliable metric extraction, proving that robust statistical methodologies can compensate for transient sensor disconnections [43].

### 4.3. Computational Constraints: Energy Consumption and Latency

A critical yet frequently overlooked challenge in deploying complex ML models (e.g., deep CNNs and Transformers) for continuous wearable monitoring is the severe hardware constraint of edge devices. While cloud-computing resolves processing power limitations, it introduces substantial latency and continuous high energy consumption for wireless data transmission, draining wearable batteries rapidly. Therefore, the transition from sensor technologies to ML algorithms must be a joint optimization process. Recent approaches, such as the Transposed Projection-CNN (TP-CNN) utilized by Zhang et al. [44], address this by executing algorithms directly on compressed ECG signals at the edge. By utilizing a 10-fold compression ratio, this approach drastically reduces energy consumption during data transmission while maintaining a 99.32% accuracy for AF detection, illustrating that optimizing computational weight and latency is as critical as raw predictive accuracy in real-world implementations.

Furthermore, direct performance comparisons across the literature must be interpreted with extreme caution. Extremely high metrics (e.g., AUC > 0.95 or F1-scores > 0.98) reported in preliminary studies often result from internal cross-validation on highly controlled, small-scale datasets (*n* < 50) without external validation cohorts. This raises significant concerns regarding subject-level data leakage and model drift when applied to heterogeneous ambulatory environments.

## 5. Heart Rate Variability (HRV) as a Core Digital Biomarker

HRV—the physiological phenomenon of variation in the time interval between consecutive heartbeats—is heavily modulated by the sympathetic and parasympathetic branches of the autonomic nervous system, making it an invaluable window into a patient’s internal neurophysiological and psychological state. A detailed synthesis of these HRV metric associations in cognitive and psychiatric disorders is provided in Table 2.

### 5.1. HRV in Mental Health and Psychiatric Disorders

Wearable-derived HRV is widely investigated as an objective proxy for psychological stress, anxiety, and depression [52,53]. In a massive longitudinal analysis comprising over 181,574 individuals observed over 13 months (accumulating 7,942,176 days of total wear time), Presby et al. [54] found that higher HRV and lower resting HR were significantly associated with lower self-reported levels of depression, anxiety, and stress. Cross-lagged models confirmed that higher levels of reported stress longitudinally preceded lower HRVs, higher respiratory rates, and higher resting HR [54]. Importantly, while traditional sleep metrics like sleep efficiency and total sleep time often show no statistical association with generalized anxiety in meta-analyses, elevated HR and diminished physical activity remain robust, actionable indicators [7].

Models utilizing digital phenotyping have demonstrated impressive predictive clinical capabilities. Zhan et al. [45] conducted a 12-week prospective observational study with 183 participants diagnosed with major depressive disorder (MDD) initiating standard treatment. By leveraging an RF model incorporating 14 digital features—including reduced geographic mobility, decreased social app usage, and disturbed sleep patterns—they achieved a 76.4% accuracy (sensitivity 73.1%, specificity 79.6%) in predicting individual treatment response [45]. Another study aiming to predict mood symptoms in bipolar disorder (BD) achieved an 83% accuracy (area under the receiver operating characteristic curve [AUROC] 0.89) for depressive symptoms and 91% accuracy (AUROC 0.88) for manic symptoms using wearable digital biomarkers processed through ML algorithms like extreme gradient boosting (XGBoost). The model utilizing explainable AI revealed that high resting HR, low activity, and lack of sleep accurately predicted depressive episodes [47].

Advanced deep learning applications analyzing ultra-short R-R intervals have also proven effective in the automatic assessment of severe psychiatric illnesses. Książek et al. [48] demonstrated that analyzing 1- to 5-min ECG windows using low-cost wearables yielded 83% classification accuracies for schizophrenia and BD via SVM and XGBoost, a performance comparable to advanced, high-cost clinical diagnostic methods [48]. Furthermore, Kiguchi et al. [46] successfully utilized XGBoost models on smartphone and wearable data to predict symptom worsening in remitted depression patients on maintenance pharmacotherapy, achieving a weighted kappa of 0.68 by incorporating lifestyle patterns (e.g., participants spending more time indoors with unstable sleep versus those spending more time working/studying) into the predictive matrix. Studies assessing continuous anxiety monitoring also confirm that deep bidirectional-LSTM networks trained heavily on HRV can identify distinct emotional states (neutral, happy, sad) with up to 97% accuracy [49].

### 5.2. Environmental, Contextual, and Exertional Modulators of HRV

HRV is highly responsive to external stimuli, environment, and physical exertion. Studies investigating the effect of music on HRV revealed that slow, classical music and white noise significantly enhance HRV metrics (e.g., root mean square of successive differences [RMSSD], standard deviation of normal-to-normal intervals [SDNN], and high-frequency power [HF]) compared to electronic or personal music. White noise specifically showed the greatest increase in HRV, hypothesized to induce a relaxation response and increased parasympathetic activity due to its consistent and monotonous sound characteristics [55]. Furthermore, environmental stressors such as prolonged cold-air exposure (10 °C) have been shown to deteriorate cognitive performance (significantly impairing short-term memory and reaction time). This cognitive decline can be accurately classified (82.4% accuracy, 84.2% sensitivity) using HRV features extracted from wearable ECGs, significantly outperforming EDA metrics which only yielded 55.4% accuracy [56].

Short-term detection of dynamic stress levels has also been validated in exergaming and cognitive-motor tasks designed to simulate rehabilitation, proving the feasibility of unobtrusive stress monitoring in interactive environments with an overall accuracy of 0.70 across four stress levels [57]. Dedicated wrist systems employing mid-level physiological fusion have further corroborated the predictive power of daily stress monitoring utilizing HRV combined with peripheral capillary oxygen saturation (SpO2) and skin temperature, where temperature and HRV showed the strongest correlations with simulated stress levels (−0.43 and 0.36, respectively) [58].

## 6. Multimodal Biomarkers: Bridging the Gap Between Physiology and Biochemistry

**Figure 3:**
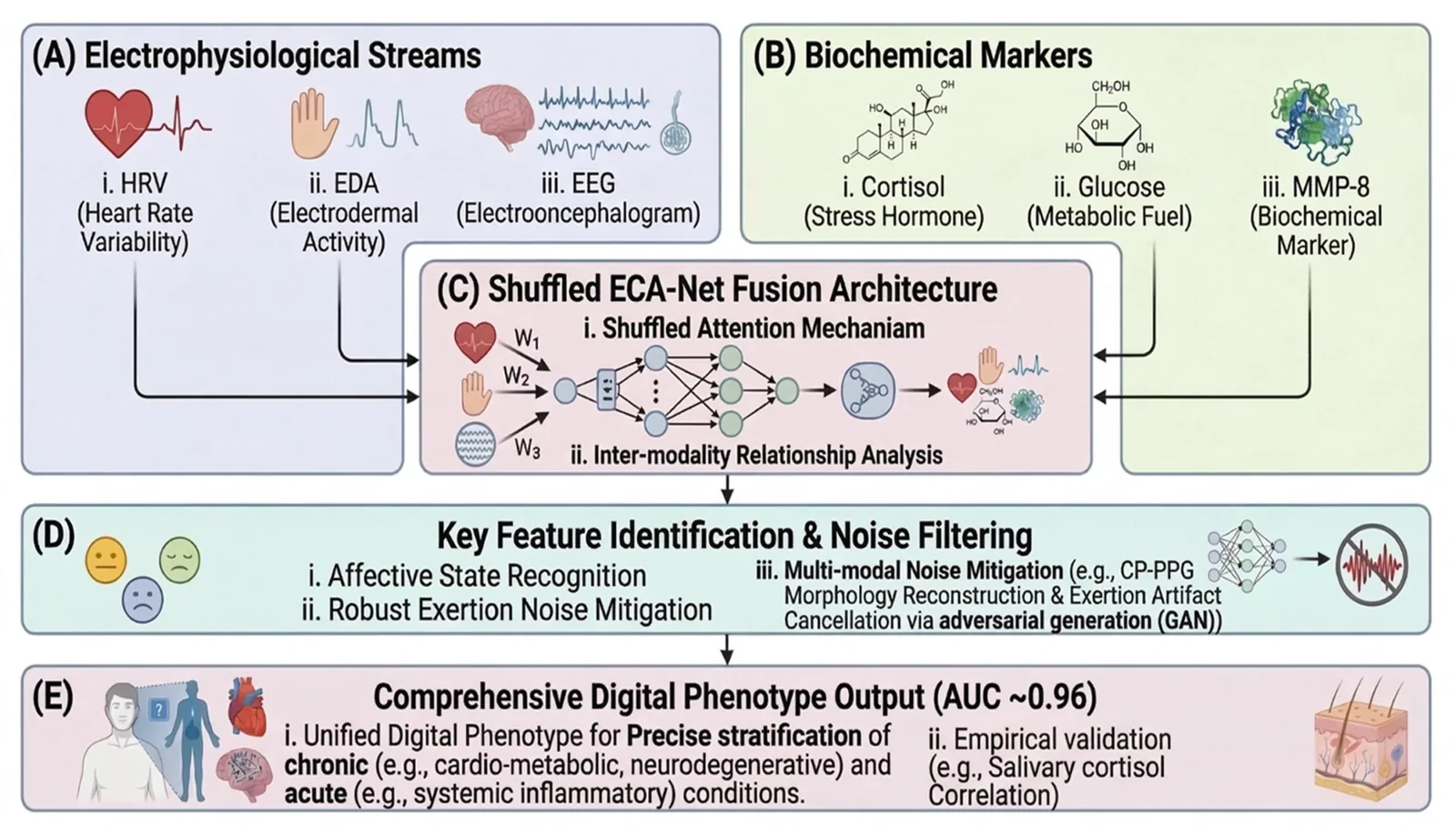
**Cyber-Physiochemical Interface: Multimodal Sensor Fusion.** Table deep learning-based cyber-fusion architecture integrating electrophysiological and biochemical streams. (**A**) **Electrophysiological Streams.** Wearable biosensors capture HRV, EDA, and EEG data. (**B**) **Biochemical Markers.** Tracks molecular analytes (e.g., Cortisol, Glucose, MMP-8). (**C**) **Shuffled ECA-Net Fusion Architecture.** Data feeds into a dynamic fusion hub where an “ECA-Net” deep generative model executes a “Shuffled Attention Mechanism” to adaptively weigh features and model inter-modality dependencies. (**D**) **Key Feature Identification & Noise Filtering.** Robust exertion noise mitigation applies GANs to cancel activity-induced artifacts. (**E**) **Comprehensive Digital Phenotype Output (AUC ~ 0.96).** The unified digital twin accurately stratifies chronic and acute conditions, carefully validated against true biochemical measurements. The arrows indicate the directional flow of data integration and processing. The ellipsis within the neural network diagram in (**C**) represents multiple hidden layers. Different background colors are used to visually distinguish the distinct data domains and algorithmic stages.

While unimodal sensing offers significant foundational data, the rigorous integration of multiple disparate ambulatory streams is highly critical for enhancing diagnostic precision, contextual awareness, and model generalizability across broad pathology domains. Figure 3 elucidates the detailed systematic integration framework of the ‘Cyber-Physiochemical Interface,’ illustrating how continuous electrophysiological and multi-scale molecular biochemical streams are rigorously converged via deep learning-based cyber-fusion into a unique digital phenotype for proactive care stratification.

### 6.1. Fusing Behavioral, Physiological, and Biochemical Data

The concept of a “cyber-physiochemical interface” allows for the simultaneous assessment of biomechanical movements and molecular biomarkers, creating a holistic digital twin of the patient [59]. Evaluating mental stress solely through HR can easily be confounded by physical activity or ambient temperature. A multimodal approach integrating HRV, EDA, skin temperature, and biochemical markers like salivary cortisol, glucose, or alpha-amylase provides a comprehensive, highly accurate physiological profile suitable for clinical use [34,60]. Research utilizing a comprehensive ML pipeline on data from 17 participants has shown that fusing multimodal data (EDA, ECG, and EEG) via Chi-squared (Chi2) feature selection increases stress classification accuracy by 12.9%, reaching up to 95.9% compared to unimodal approaches, identifying key biomarkers like EEG beta-to-alpha ratios and ECG root-mean-square metrics [34].

Fusing biomechanical tracking with biochemical analysis (e.g., lactate, glucose, uric acid in sweat) through novel VCFs@Ag (viscose fabrics coated with silver) interfaces drastically enhances the simultaneous recognition of muscle strain and metabolic output during exercise. Encapsulated by multi-microporous membranes (M-MPM), this system exhibits directional sweat transport while resisting pollutant interference, increasing the accuracy of multimodal recognition to 88.6% via a bidirectional memory network [61]. Similarly, combining physical activity tracking with CGM data via an ensemble feature selection-based LightGBM algorithm has enabled precise, non-invasive interstitial glucose prediction. This algorithm achieved a RMSE of 18.49 mg/dL and a MAPE of 15.58%, entirely omitting the need for manual food logs. This paves the way for personalized low-glycemic diets to manage pre-diabetes and migraines without the need for invasive, high-cost CGM devices [36].

### 6.2. Deep Learning for Multi-Modal Sensor Fusion

Advanced deep learning architectures maximize the clinical benefits of sensor fusion by heavily considering inter-modality relationships. By combining ECGs, respiratory waveforms, and electrogastrograms, models utilizing frameworks like the Shuffled ECA-Net (efficient channel attention) have achieved a psychological stress detection accuracy of 0.916, a sensitivity of 0.917, and an AUROC of 0.964. This model was rigidly validated using salivary cortisol tests as the ground truth across 26 participants, proving that combining multiple bio-signals with shuffled attention modules resolves stress-related ambiguity [50].

Furthermore, AI-enabled microfluidic platforms are rapidly expanding into infection monitoring, analyzing microbial nucleic acids, metabolites, and host immune mediators directly from sweat, saliva, tears, or wound exudates, adhering closely to advanced mechanomedicine principles [27]. Such platforms often utilize smartphone-integrated optics for decentralized diagnostics; for example, Ingole et al. [20] developed a stereolithography (SLA) 3D-printed microfluidic flow cell, processing 100 μL of sample-reagent mixture via a peristaltic pump. Imaged within a controlled lighting enclosure across multiple smartphone models, this system quantitatively estimated liver biomarkers (direct/total bilirubin ranging 0.1–20 mg/dL, alanine aminotransferase [ALT] and aspartate aminotransferase [AST] ranging 10–300 U/L) using a CNN. This clinical-grade analyzer achieved an average coefficient of determination (R^2^) of 0.997 and repeatabilities with coefficients of variation under 3%, ideal for rural healthcare and decentralized liver function assessment [20].

## 7. Clinical Applications in Chronic, Neurological, and Infectious Diseases

Digital biomarkers are rapidly transitioning from consumer wellness tracking to rigorous clinical applications, prognostic models, and FDA-cleared diagnostics that significantly impact patient care. However, it is crucial to explicitly distinguish that many of the reviewed studies concern DTx-adjacent monitoring, diagnosis, or clinical decision support rather than direct, evidence-based therapeutic interventions (DTx). The diagnostic accuracy across these varied pathologies is carefully summarized in Table 3.

### 7.1. Cardiovascular Disease and Atrial Fibrillation (AF)

Early detection of cardiac anomalies is a primary focus of mHealth. Algorithms applying ML to two-lead Holter ECGs or wearable PPG signals can accurately predict the short-term onset of paroxysmal AF before clinical manifestation. Grégoire et al. [8] trained XGBoost decision tree models on HRV parameters derived from 95,871 Holter recordings across 872 patients, capturing 1319 episodes of paroxysmal AF. They achieved an incredible AUROC of 0.919 and a positive predictive value (PPV) of 90.2% for predicting AF episodes lasting more than 5 min. This finding strongly supports the viability of targeted ‘pill-in-the-pocket’ intervention strategies hours prior to AF onset [8]. Transfer learning has also enabled gold-standard ECG trained models to seamlessly adapt for non-ambulatory AF detection using consumer-grade PPG wearables. Utilizing a one-dimensional deep CNN, models achieved 95.10% accuracy, 94.60% sensitivity, and 95.20% specificity across unseen PPG datasets [65]. Another study utilizing a continuous 24-h mHealth patch for AF screening via AI arrhythmia analysis achieved a remarkable subject-based detection accuracy of 97.2%, a sensitivity of 100%, and a specificity of 94.9% amongst 178 patients recruited from emergency care [14].

Furthermore, dynamic HRV monitoring has been leveraged to predict New York Heart Association (NYHA) functional class improvements in acute heart failure patients. Shi et al. [66] established that dynamic changes in HRV parameters between admission and discharge (particularly SDNN and SD2) serve as highly sensitive, non-invasive biomarkers for real-time treatment response assessment. RF models achieved an area under the curve (AUC) of 0.77 to prioritize high-risk patients for early intervention and reduce readmission rates [66]. Wearable monitoring in ambulatory heart failure patients is further being validated through extensive 1-year prospective tracking via CATCH-ECG protocols to develop predictive algorithms utilizing cloud-based mobile apps [67]. The broader implications of AI in cardio-oncology are also being heavily realized, integrating wearable data, ECGs, imaging, and circulating serum biomarkers to predict, stratify risk, and longitudinally manage cardiotoxicity related to complex cancer therapies [68]. Deep learning methods like the transposed projection-convolutional neural network (TP-CNN) have even allowed for AF detection directly on compressed ECG signals in the cloud. By utilizing a simple deterministic measurement matrix (SDMM), the TP-CNN achieved 99.32% accuracy and 99.43% specificity at a 10-fold compression ratio, heavily reducing energy consumption during data transmission for wearable IoT devices [44].

### 7.2. Neurological and Movement Disorders

In neurodegenerative conditions like Parkinson’s disease (PD), digital biomarkers offer objective, high-resolution measures of disease progression, addressing severe clinical issues like freezing of gait (FOG) with expansive bibliometric deep learning analysis, where CNN architectures frequently yield accuracy and AUC metrics above 0.91 [69]. Federated learning frameworks integrating EEG and resting-state functional MRI (rs-fMRI) data have vastly improved early-stage PD diagnosis while simultaneously preserving patient privacy through decentralized, dynamic model aggregation, yielding improved receiver operating characteristic area under the curve (ROC-AUC) scores tailored to demographic-aware profiles [70]. Wearable sensors capturing gait parameters and temporal variability have been utilized to classify PD severity subtypes reliably. Park H. et al. [16] demonstrated that using specific principal components of bilateral ankle kinematics derived from wearable motion sensors resulted in excellent classification performance (AUC of 1.0), easily distinguishing clinically severe from mild subtypes of PD in a cohort of 102 patients [16]. Similarly, an integrative approach leveraging PPG, HRV, and 6-axis IMUs to detect tremor amplitudes, rigidity, and bradykinesia achieved over 96.7% accuracy utilizing an ensemble multi-layer perceptron model in identifying early-stage PD and atypical Parkinsonian syndromes like multiple system atrophy (MSA) and progressive supranuclear palsy (PSP) [17].

Wearable-based physiological measures, particularly HRV, have also shown strong correlations with executive function in patients with mild cognitive impairment (MCI) and early dementia, facilitating unobtrusive, remote mental health tracking. A 10-week clinical trial leveraging a self-supervised convolutional autoencoder demonstrated that digital biomarkers (combining HRV and skin conductance) predicted daily depression severity scores with a correlation of r = 0.73, and total severity of mood disorder symptoms with r = 0.67 in older adults [51]. In pediatric neurodevelopment, smartwatch-based ML models utilizing HR, sleep, and motor activity conditional dependencies via decision trees achieved 80.89% accuracy in predicting severe disruptive behavioral outbursts in hospitalized children aged 7–10 years [71]. Moreover, full-body motion tracking suits integrated with ML accurately predicted longitudinal disease trajectories in Duchenne muscular dystrophy, outperforming standard clinical assessments [72]. In Friedreich’s ataxia, where current trials rely on simplistic behavioral assessments, digital behavioral features (kinematics from the 8-m walk and 9-hole peg test) predicted personal clinical scores 1.7 to 4 times more precisely than standard clinical scales (spinocerebellar ataxia functional index [SCAFI] and scale for the assessment and rating of ataxia [SARA]) 9 months into the future, and accurately predicted FXN gene expression levels [73]. Complex symptom progression in rare disorders like Rett syndrome is also being tracked via sensor-driven EDA, max HR percentages, and HR/low-frequency (LF) ratios to precisely capture physiological morbidities such as epilepsy and gastrointestinal distress [74].

### 7.3. Predicting Systemic Inflammatory Responses and Infection

Digital biomarkers are proving exceptionally vital in predicting systemic inflammatory risks and post-operative recovery. Multimodal models integrating continuous wearable-based behavioral lifelogs (step count, sleep efficiency) with physiological data (HRV, SpO2) successfully predicted ≥ 1.0 mg/L reductions in high-sensitivity C-reactive protein (hs-CRP) in post-percutaneous coronary intervention (PCI) patients. Transformer models yielded an AUC of 0.88 and an F1-score of 0.81, vastly outperforming standard logistic regression models and confirming the strong predictive contribution of modifiable behavioral features over a 6-month monitoring period [62]. Furthermore, innovative diagnostic algorithms utilizing traditional CRP metrics via digital clinical decision support systems are being tested in rigorous randomized controlled trials to safely guide and reduce antimicrobial therapy durations without compromising patient safety [75].

In the realm of infectious diseases, ML applied to wearable data (HR, breathing rate, acceleration via smart rings and shirts) predicted systemic inflammatory surges following a controlled live attenuated influenza vaccine challenge with an ROC-AUC of 0.91 and specificity of 0.83, vastly outperforming traditional symptom-based detection methods (ROC-AUC 0.79) in a cohort of 56 participants tracked over 12 days [63]. HRV metrics processed via ML have also been successfully utilized to classify individuals with active COVID-19 and post-COVID-19 conditions. Sanches et al. [76] found that participants with active COVID-19 showed significantly lower HRV indexes (SDNN, RMSSD, LF%, HF%) compared to healthy controls (*p* < 0.001). Using decision tree models, they achieved an 87% overall classification accuracy (F1-score 92% for post-COVID-19) when a clinical contextual variable indicating recent SARS-CoV-2 infection was included alongside the HRV features [76].

AI-driven audio and vocal biomarkers are also emerging rapidly as promising tools for detecting cognitive decline, monitoring respiratory diseases (asthma, chronic obstructive pulmonary disease [COPD], pneumonia), and predicting fatigue levels in long-COVID-19 patients. A systematic review revealed that audio biomarkers achieved 80–100% accuracies utilizing artificial neural networks on breathing and coughing samples [21]. Models combining logistic regression, k-nearest neighbors, and SVM trained on 1772 voice recordings tracked over 2 weeks successfully discriminated COVID-19 participants with fatigue, achieving an AUC of up to 86% across smartphone operating systems [22]. Additionally, scoping reviews are establishing protocols to synthesize AI-driven audio biomarkers for detecting Alzheimer’s, dementia, and depression in geriatric populations [23]. In low- and middle-income countries (LMICs), continuous wearable monitoring of HRV linked with recurrent neural networks outperformed traditional bedside monitors in predicting sepsis mortality among 50 hospitalized patients (AUC = 0.83), showcasing the immense global health utility and cost-effectiveness of these affordable digital tools [77,78].

## 8. Sleep, Exertion, and Behavioral Phenotyping

DTx and adjacent diagnostic technologies extensively leverage wearable data to phenotype behaviors related to sleep architecture, physical exertion, ovulation cycles, and overall lifestyle tracking, allowing for highly precision-guided lifestyle interventions.

### 8.1. Sleep Staging, Apnea, and Reproductive Health

Polysomnography (PSG) remains the clinical gold standard for sleep analysis, but patch-type wearable ECG and impedance pneumography (IPG) offer highly scalable, in-home alternatives that eliminate the cost and impracticality of routine PSG. Deep learning models evaluating IPG and HRV metrics (from 5-min windows) have achieved 83.6% accuracy (AUROC: 86.0%) in binary (Wake/Sleep) classifications and robust F1-scores in multi-stage (Wake, REM, N1, N2) classifications. Feature reduction techniques utilizing mRMR identified the top 15 features that preserved 99% of classification performance while heavily reducing training time by 73% [79]. The use of fuzzy recurrence plots (FRPs) to visually transform one-dimensional (1D) HRV time series into two-dimensional (2D) textures during relaxation and sleep further aids in automated detection, achieving 96.6% accuracy and 100% specificity via SVM classifiers utilizing only three selected features [80].

Innovative approaches to pediatric obstructive sleep apnea (OSA) highlight the critical integration of AI, biomarkers, 3D facial photography, and wearable technology to bypass the severe limitations of limited diagnostic PSG availability in children, offering promising alternatives for early detection [81]. Moreover, ML models predicting ‘good sleep’ (defined as ≥ 90% sleep efficiency) improved significantly (AUC = 0.80, accuracy = 0.85) when 2-day combined physical activity, light exposure, and heart rate variability data were utilized within an XGBoost framework across 2136 nights of data [82]. In the realm of women’s health, LightGBM frameworks generating novel features by integrating temporal HRV patterns with continuous temperature values during sleep achieved an AUROC of 0.84 in predicting ovulation windows accurately up to 5 days in advance for women with highly irregular cycles, significantly enhancing fertility management [83].

### 8.2. Fatigue, Exertion, and Wearable Analytics

Accurately assessing physical exertion and fatigue is crucial for rehabilitation, sports medicine, occupational health, and predicting preoperative cardiorespiratory fitness (CRF). Hussain et al. [84] employed a ML ensemble regression model (RF) utilizing wearable-derived moderate-to-vigorous physical activity (MVPA), maximum activity energy expenditure (aEEmax), and non-linear HRV to predict CRF index scores (e.g., 6-min walk test distances) with an R^2^ of 0.91 prior to major noncardiac surgery [84]. Deep learning approaches, such as LSTM with Multi-Head Attention, have successfully classified rating of perceived exertion (RPE) utilizing continuous inputs of HRV, SpO2, and pedal speed during cycling exercises. Analyzing segmented 2-min intervals, the LSTM model achieved an impressive 82.9% accuracy and an F1 score of 86.3% [38].

In clinical settings, ultra-short-term ECG measurements analyzed via complex CNN architectures (Deep ECGNet) accurately predicted mental fatigue and stress states without relying on traditional, computationally heavy feature engineering. Utilizing optimal convolution filter lengths related to P, Q, R, S, and T wave durations, the model improved accuracy by up to 16.22% over conventional HRV methods [64]. MyWear systems, utilizing Internet of Things (IoT) T-shirts embedded with continuous physiological monitoring, demonstrated how multi-sensor data processed via SVM can achieve 98% accuracy in identifying irregular heart rhythms and tracking stress dynamically [85]. Furthermore, behavioral adherence itself plays a massive role in physiological outcomes; a monumental longitudinal study of 11,914 subscribers leveraging nearly a million days of data revealed that wearing devices like WHOOP more frequently was directly associated with better biometrics (lower resting HR, higher HRV), longer and more consistent sleep duration, and greater weekly physical activity duration, emphasizing the physiological and behavioral benefits of consistent DTx engagement [86].

## 9. Challenges, Limitations, and Future Directions

**Figure 4:**
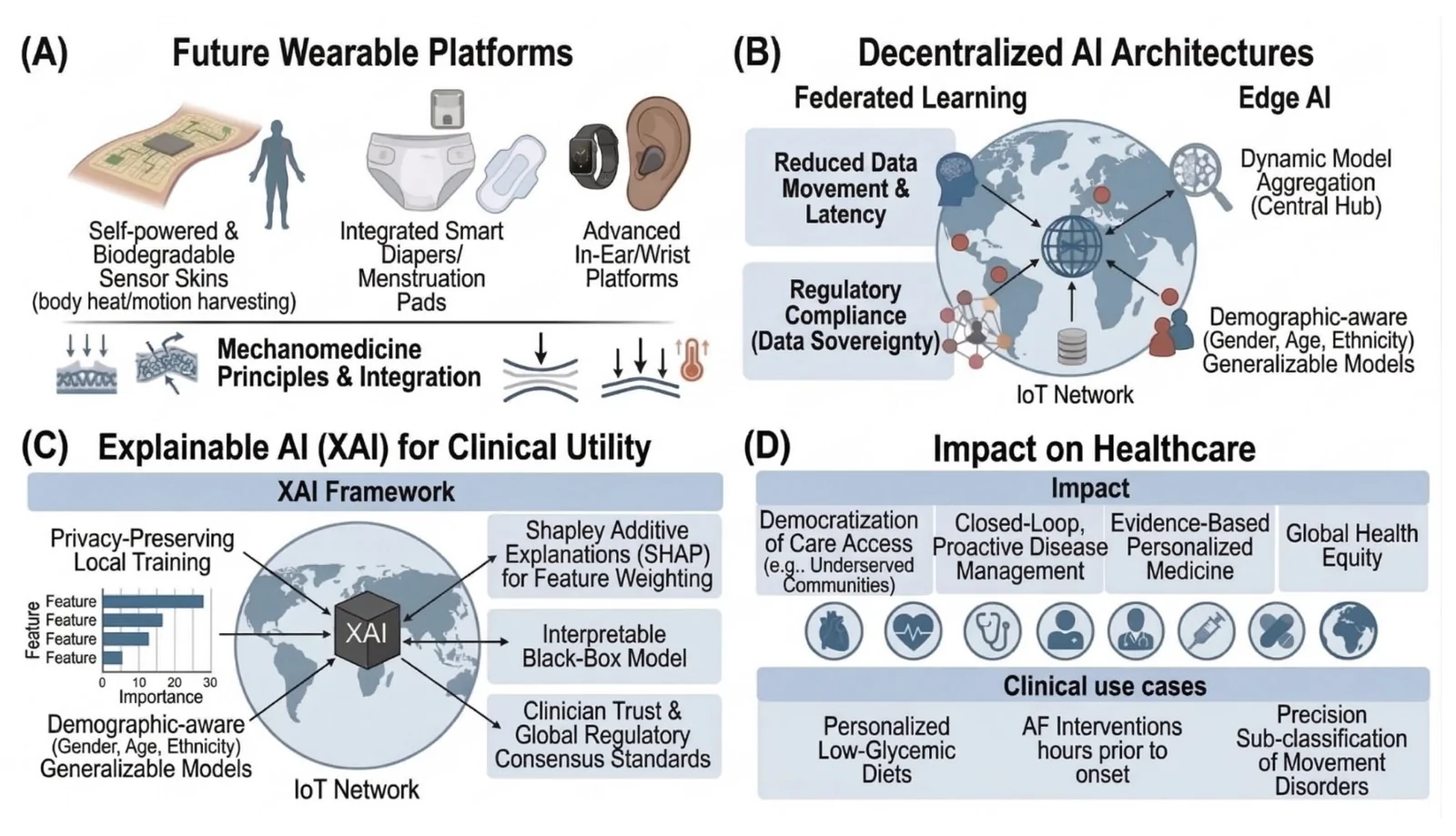
**Future Trajectories in Digital Therapeutics & Advanced Wearables.** A diagram illustrating future directions required to advance digital therapeutics, focusing on the convergence of next-generation hardware, decentralized processing, and interpretable models to achieve global health equity. The framework is divided into four sections: (**A**) **Future Wearable Platforms.** Integration of mechanomedicine principles into unobtrusive hardware (e.g., biodegradable skins, microfluidic pads). (**B**) **Decentralized AI Architectures.** Contrasts Federated Learning (ensuring data sovereignty and reduced latency via central aggregation) with Edge AI (optimizing demographic-aware local models to prevent bias). (**C**) **Explainable AI** (**XAI**) **for Clinical Utility.** Employs Shapley Additive exPlanations (SHAP) to translate complex “black-box” predictions into explicit feature weighting, establishing clinician trust. (**D**) **Impact on Healthcare.** Broadening of access and closed-loop proactive management for precision medicine, highlighting clinical use cases like AF intervention prior to onset. The arrows indicate the directional flow of data and technological integration across the networks, while the distinct background colors are used to visually separate the four key thematic sections.

While the integration of these technologies heralds a new era in personalized medicine, several critical informatics and computational limitations persist both within the current literature and this review. Addressing these structural data silos, algorithmic biases, and processing challenges is essential before these digital pipelines can be reliably integrated into interoperable electronic health records (EHRs) and clinical decision support systems. A detailed breakdown of these methodological limitations and proposed algorithmic mitigations is listed in Table 4. Additionally, an overview of future trajectories addressing these challenges is conceptualized in Figure 4.

### 9.1. Methodological and Technical Limitations

A significant barrier consistently identified in the current literature is the heavy reliance on small sample sizes, varying data quality, and a distinct lack of standardized protocols across studies [35,89]. Consumer wearables operating in unpredictable ambulatory environments often suffer from missing data due to poor wear compliance, motion artifacts, or battery limitations. Studies systematically testing data degradation demonstrate that while basic metrics like the median interbeat interval (IBI) and standard deviation of IBI (STDRR) remain relatively stable until up to 60% and 35% data degradation at rest, highly sensitive frequency-domain measures (e.g., LF/HF ratios) become entirely unstable and invalid with merely 10% missing data [43]. Consequently, rigorous data cleaning, continuous cross-validation, and the global harmonization of ultra-short HRV feature extraction algorithms are imperative to avoid performance inflation and algorithmic shortcut learning [40,90]. Vos et al. [89] highlighted that only 54% of studies utilizing low-cost EEG devices included health screening prior to experimentation, and the lack of consensus on data pre-processing and sensor placement severely limits model generalization [89].

### 9.2. Generalizability, Bias, and Privacy

ML models trained on specific, homogeneous datasets or singular geographical cohorts often fail completely to generalize across broader, diverse populations, a phenomenon known as “overfitting” [87]. Studies frequently highlight the pressing need for demographic-aware frameworks to address gender-specific, age-specific, or ethnicity-specific biomarker differences, ensuring equitable healthcare. For example, Pratihar & Sankar [70] found that gender-specific evaluations in PD detection suggested male-exclusive models currently outperform female models in biomarker representation, indicating a severe need for balanced training sets [70].

The integration of continuous physiological monitoring into clinical pathways must also carefully navigate stringent patient privacy concerns (e.g., Health Insurance Portability and Accountability Act [HIPAA], General Data Protection Regulation [GDPR] compliance), complex data interoperability standards across electronic health records (EHRs), and the perceived psychological intrusiveness of continuous monitoring. A large international mixed-methods study comprising 1010 participants from 30 countries confirmed that patients perceived remote digital monitoring as highly intrusive particularly when it involved private-sector data handling (β = −0.15 compared to public-sector), invasive food monitoring, or real-time physician-generated human feedback. Four drivers of intrusiveness emerged: practical/psychosocial burden, control loss, data misuse fears, and the dehumanization of care, revealing critical behavioral barriers to long-term adoption [88].

### 9.3. Future Trajectories

Future directions point toward advanced biomedical informatics architectures, particularly decentralized machine learning paradigms like federated learning. By training models locally on edge client nodes while aggregating insights centrally, these architectures preserve patient data sovereignty and comply with stringent privacy frameworks without sacrificing model accuracy [70]. For instance, Pratihar & Sankar [70] successfully implemented a decentralized FL framework for Parkinson’s disease diagnosis across multiple institutions. By aggregating weights derived from local EEG and rs-fMRI data without centralizing the raw imaging or electrophysiological files, they dynamically adjusted model parameters to achieve high diagnostic accuracy while maintaining strict compliance with GDPR and HIPAA regulations. This approach crucially mitigates dataset shift and enhances generalizability across diverse geographic cohorts without compromising patient sovereignty. Concurrently, innovations in material informatics will likely yield biodegradable, self-powered sensing platforms that conform seamlessly to the skin, eliminating battery constraints and enabling uninterrupted continuous data pipelines [10,61].

Additionally, integrating complex digital biomarkers with explainable artificial intelligence (XAI) frameworks—such as LivXAI-Net, which utilizes Shapley additive explanations (SHAP) values to interpret key biochemical features—will effectively bridge the gap between impenetrable “black-box” algorithms and transparent clinical decision support systems [84,91]. The application of these advanced computational models is rapidly expanding across complex informatics domains. Future research must optimize these pipelines to facilitate telemedicine data streams and algorithmic risk stratification in predicting substance use disorder relapse [92], personalized oncology via diagnostic hubs (e.g., predicting targeted treatments for Head and Neck or breast cancer cohorts) [93,94], and optimizing clinical decision support systems for population-level metabolic tracking like Type 2 Diabetes [95].

## 10. Conclusions

The final conclusion of this structured narrative review asserts that while digital biomarkers possess the computational potential to rival traditional clinical diagnostics, their true scalability is currently bottlenecked by informatics challenges: fragmented data silos, artifact susceptibility, and algorithmic bias. The ultimate clinical relevance of this digital revolution lies in building robust, interoperable data architectures that shift the paradigm from episodic, reactive treatments to continuous, proactive, and highly tailored monitoring.

By expertly developing machine learning pipelines that translate raw, high-dimensional physiological and biochemical data—particularly HRV, microfluidic analytics, and kinetic gait parameters—into actionable and reliable digital phenotypes, informaticians can provide healthcare providers with highly transparent clinical decision support. These pipelines serve as a crucial foundation for both DTx-adjacent diagnostics and future evidence-based therapeutic interventions. While algorithmic biases, signal artifact susceptibility, and stringent data privacy protocols remain essential computational challenges, the ongoing evolution of multimodal sensor fusion algorithms, explainable AI (XAI), and demographic-aware computing models promises to establish digital biomarkers as a reliable pillar of precision healthcare. Moving forward, the biomedical informatics community must rigorously prioritize the establishment of global data interoperability standards, the deployment of decentralized federated learning networks, and the creation of highly inclusive training datasets to ensure generalizable and equitable algorithmic performance.

## Figures and Tables

**Table 1:** **Primary Wearable Sensor Modalities and Target Biomarkers.**

Sensor Modality	Primary Biological Target	Extracted Digital Biomarker	Target Pathologies	Research Model/Device	Clinical & Statistical Significance
**PPG**	Blood volume changes	HR, HRV	Stress, Drowsiness	Smartwatches, Earbuds, CP-PPG	Deep generative models (CP-PPG) improved signal fidelity by 40% (MAE: 0.09), rescuing HRV metrics from severe motion artifacts [11,12].
**ECG** **/Patch**	Cardiac electrical activity	R-R Intervals, HRV, Arrhythmia events	Heart Failure, AF, Ischemic Stroke,	Continuous mHealth Patch, In-ear ECG	High precision enables AF detection with subject-based accuracy of 97.2% and 100% sensitivity. Single-ear ECG achieved 0.49 bpm HR error at 7.6 mW power [8,13,14].
**IMU**	Biomechanical movement	Gait speed, GSG, Tremor amplitude	PD, Frailty, Fall Risk	Wrist-worn gyroscopes, Bilateral ankle sensors	Captures temporal variability in gait; capable of classifying PD severity subtypes with excellent accuracy (AUC 1.0) utilizing ankle kinematics [15,16,17].
**Microfluidic Sensors**	Electrolytes, Metabolites	Cortisol, MMP-8, Glucose, Liver Enzymes	Periodontal Inflammation, Wounds, Liver Disease	Cactus-inspired Janus membranes, 3D-printed flow cells	AI-assisted smartphone flow cells achieved R^2^ of 0.997 for liver biomarker quantification. Janus membranes improve biosafety by isolating chemical indicators [18,19,20].
**Acoustic/Audio Sensors**	Vocal/respiratory patterns	Vocal pitch, Breathing rate, Cough frequency	COPD, Asthma, COVID-19 Fatigue, Cognitive Decline	Smartphone microphone arrays	Novel digital biomarker; effectively monitors respiratory distress and cognitive fatigue, reliably discriminating COVID-19 fatigue with an AUC up to 86% across operating systems [21,22,23].

***Note*: PPG**: Photoplethysmography, **HR**: Heart Rate, **HRV**: Heart Rate Variability, **AF**: Atrial Fibrillation, **CP-PPG**: Contact-Pressure mitigated PPG, **MAE**: Mean Absolute Error, **ECG**: Electrocardiogram, **mHealth**: Mobile Health, **IMU**: Inertial Measurement Unit, **GSG**: Gyrosphygmogram, **PD**: Parkinson’s Disease, **AUC**: Area Under the Curve, **MMP-8**: Matrix Metalloproteinase-8, **AI**: Artificial Intelligence, **COPD**: Chronic Obstructive Pulmonary Disease.

**Table 2:** **HRV and Behavioral Metric Associations in Psychiatric and Cognitive Disorders.**

Psychiatric/Cognitive Condition	Primary HRV/Digital Metrics	Algorithm Used	Study Duration & Sample	Statistical Outcome	Physiological & Clinical Implication
**MDD**	Geographic mobility, social app usage, sleep patterns, lifestyle patterns	RF, XGBoost	12-week prospective; *n* = 183	76.4% accuracy (Sensitivity 73.1%, Specificity 79.6%)	Predicts individual treatment response and identifies symptom worsening in remitted patients on maintenance pharmacotherapy via lifestyle tracking [45,46].
**BD**	Resting HR, Activity proxies, Sleep deprivation	XGBoost	Cross-sectional; *n* = 24	83% accuracy (AUROC 0.89) for depressive; 91% (AUROC 0.88) for manic	High resting HR combined with low activity and lack of sleep strongly predicts mood episodes via explainable AI, enabling timely interventions [47].
**Schizophrenia Spectrum**	Unprocessed R-R intervals (HRV)	Adaptive LSTM, SVM	7–113 days; *n* = 37	Mean F1 score of 0.9817	Predicts psychosis relapses directly from raw HRV data; eliminating artifact preprocessing improved model learning from temporal physiological trends [41,48].
**Psychological Stress/Anxiety**	HRV, EDA, EEG, Skin Temperature	Shuffled ECA-Net, Chi2, bi-LSTM	Ambulatory continuous; *n* = 17 to *n* = 26	Accuracy up to 97%	Fusing multimodal data (e.g., EEG and ECG root-mean-square) minimizes false positives caused by physical exertion [34,49,50].
**MCI**	HRV, Skin Conductance	Convolutional Autoencoder	10 weeks; *n* = 30	Correlation r = 0.73 (depression severity), r = 0.67 (mood)	HRV features highly correlated with executive function deterioration in older adults, offering remote mental health tracking without repetitive testing [51].

***Note*: HRV**: Heart Rate Variability, **MDD**: Major Depressive Disorder, **RF**: Random Forest, **XGBoost**: Extreme Gradient Boosting, **BD**: Bipolar Disorder, **HR**: Heart Rate, **AUROC**: Area Under the Receiver Operating Characteristic Curve, **LSTM**: Long Short-Term Memory, **SVM**: Support Vector Machine, **EDA**: Electrodermal Activity, **EEG**: Electroencephalogram, **ECA-Net**: Efficient Channel Attention, **Chi2**: Chi-squared, **bi-LSTM**: Bidirectional Long Short-Term Memory, **ECG**: Electrocardiogram, **MCI**: Mild Cognitive Impairment.

**Table 3:** **Critical Comparison of ML Diagnostic Algorithms Across Pathologies.**

Pathology/Condition	Feature Set	ML Algorithm	Sample Size (*n*) & Validation Method	Statistical Outcome	Methodological Limitations & Clinical Utility
**AF**	HRV (SDNN, RMSSD)/Compressed ECG	XGBoost/Deep CNN/TP-CNN	*n* = 872 (Holter)/*n* = 178 (Patch)	AUC: 0.919; Sensitivity: 100%	Limitation: Models trained on Holter data may suffer from dataset shift when applied to noisy PPG. Utility: TP-CNN reduces energy latency via 10-fold compression [8,14,44].
**Post-PCI Inflammation**	HRV, Sleep efficiency, SpO2, Steps	Transformer Model	Not explicitly specified	AUC: 0.88; F1 Score: 0.81	Limitation: High computational cost of Transformers limits direct on-device processing. Utility: Predicts hs-CRP reductions to guide cardiovascular interventions [62].
**Influenza Inflammatory Surge**	Night-time HRV, Breathing Rate	Machine Learning	*n* = 56 (Live attenuated vaccine challenge)	ROC-AUC: 0.91; Specificity: 83%	Limitation: Controlled vaccine challenge environment may not perfectly mirror natural, spontaneous infection noise. Utility: Outperformed traditional symptom detection [63].
**Liver Disease** (**Biomarkers**)	Bilirubin, ALT, AST	CNN (Smartphone-integrated optics)	Benchtop validation	R^2^: 0.997; CV < 3%	Limitation: Requires microfluidic hardware adherence and proper lighting conditions for camera accuracy. Utility: Decentralized, clinical-grade liver function assessment [20].
**Physical Exertion/Fatigue**	HRV Time/Freq domains, SpO2, Pedal Cadence, Ultra-short ECG	LSTM with Multi-Head Attention/Deep ECGNet (CNN)	Windowed analysis during cycling	Accuracy: 82.9%; F1 Score: 86.3%	Limitation: Relying on precise sensor positioning during rigorous movement risks severe data degradation. Utility: Real-time prediction of RPE and mental fatigue [38,64].

***Note*: ML**: Machine Learning, **AF**: Atrial Fibrillation, **ECG**: Electrocardiogram, **PPG**: Photoplethysmography, **HRV**: Heart Rate Variability, **SDNN**: Standard Deviation of Normal-to-Normal intervals, **RMSSD**: Root Mean Square of Successive Differences, **XGBoost**: Extreme Gradient Boosting, **CNN**: Convolutional Neural Network, **TP-CNN**: Transposed Projection Convolutional Neural Network, **AUC**: Area Under the Curve, **PCI**: Percutaneous Coronary Intervention, **hs-CRP**: High-Sensitivity C-Reactive Protein, **ROC-AUC**: Receiver Operating Characteristic Area Under the Curve, **ALT**: Alanine Aminotransferase, **AST**: Aspartate Aminotransferase, **CV**: Coefficient of Variation, **SpO2**: Peripheral Capillary Oxygen Saturation, **LSTM**: Long Short-Term Memory, **RPE**: Rating of Perceived Exertion.

**Table 4:** **Key Technical and Methodological Limitations in Digital Biomarker Research.**

Limitation Category	Description of Issue	Statistical & Physiological Impact	Proposed Solutions/Mitigations	Impact on Generalizability
**Data Degradation & Artifacts**	Highly sensitive metrics (e.g., LF/HF ratio) become unstable and invalid with just 10% missing data due to motion [43].	Invalidates autonomic nervous system metrics; introduces heavy false-positive clinical alerts.	Implementation of deep generative models (CP-PPG) and adaptive LSTM networks robust to unprocessed R-R intervals [12,41].	High; algorithms trained exclusively on clean, clinical-grade data fail in noisy ambulatory settings.
**Sample Size & Homogeneity**	Studies frequently feature homogeneous, small subject groups drawn from narrow geographic locations.	Causes severe model overfitting, missing gender-specific or age-specific physiological baseline differences.	Utilizing Federated Learning to aggregate global data locally; ensuring balanced training sets for gender representation [70,87].	Severe; restricts the regulatory approval and safety of DTx for underrepresented populations.
**Lack of Protocol Standardization**	Disparate feature extraction windows (e.g., 30-s vs. 5-min epochs) for ultra-short HRV calculations [87].	Makes cross-study comparisons mathematically unreliable and creates conflicting baseline metrics.	Establishment of global consensus guidelines and minimum reporting datasets for digital biomarkers [3].	Moderate; creates fragmented research silos that significantly slow down clinical translation.
**Data Privacy & Intrusiveness**	Patients perceive continuous monitoring as intrusive, specifically fearing private-sector data misuse [88].	Decreased compliance shrinks data volume; stress from being monitored skews baseline physiological data.	Decentralized AI processing at the edge; ensuring transparent data governance; non-intrusive feedback loops.	High; poor patient adherence directly neutralizes the predictive power of longitudinal ML models.

***Note*: PPG**: Photoplethysmography, **LF**: Low-Frequency power, **HF**: High-Frequency power, **CP-PPG**: Contact-Pressure mitigated PPG, **LSTM**: Long Short-Term Memory, **DTx**: Digital Therapeutics, **AI**: Artificial Intelligence.

## Data Availability

No new data were created or analyzed in this study. Data sharing is not applicable to this article.
